# InSituCor: exploring spatially correlated genes conditional on the cell type landscape

**DOI:** 10.1186/s13059-025-03554-1

**Published:** 2025-04-24

**Authors:** Patrick Danaher, Dan McGuire, Lidan Wu, Michael Patrick, David Kroeppler, Haiyan Zhai, Deniz G. Olgun, Dennis Gong, Jingyi Cao, William L. Hwang, Joachim Schmid, Joseph M. Beechem

**Affiliations:** 1 Bruker Spatial Biology, Seattle, WA USA; 2 Center for Systems Biology, Massachusetts General Hospital and Harvard Medical School, Boston, MA USA; 3 Department of Radiation Oncology, Massachusetts General Hospital, Brigham and Women’s Hospital and Harvard Medical School, Boston, MA USA; 4 Center for Cancer Research, Massachusetts General Hospital, Harvard Medical School, Boston, MA USA; 5 Broad Institute of MIT and Harvard, Cambridge, MA USA; 6 Gene Lay Institute of Immunology and Inflammation, Brigham and Women’S Hospital, Massachusetts General Hospital and Harvard Medical School, Boston, MA USA; 7 Harvard-MIT Health Sciences and Technology Program, Cambridge, MA USA; 8 Department of Neuroscience, University of Virginia School of Medicine, Charlottesville, VA USA

**Keywords:** Spatial transcriptomics, Spatial correlation, Statistical methods

## Abstract

In spatial transcriptomics data, spatially correlated genes promise to reveal high-interest phenomena like cell–cell interactions and latent variables. But in practice, most spatial correlations arise from the spatial arrangement of cell types, obscuring the more interesting relationships we hope to discover. We introduce InSituCor, a toolkit for discovering modules of spatially correlated genes. InSituCor returns only correlations not explainable by already-known factors like the cell type landscape; this spares precious analyst effort. InSituCor supports both unbiased discovery of whole-dataset correlations and knowledge-driven exploration of genes of interest. As a special case, it evaluates ligand-receptor pairs for spatial co-regulation.

**Supplementary Information:**

The online version contains supplementary material available at 10.1186/s13059-025-03554-1.

## Background

Single-cell spatial transcriptomics data, in which hundreds to thousands of genes are measured in situ across potentially millions of cells, poses a daunting version of the central challenge of ‘omics: how can an analyst possibly discover all the interesting biology contained within a dataset? One class of exploratory analyses looks for spatially correlated sets of genes; that is, genes that tend to be expressed in the same regions. Spatial correlation between genes can arise through direct cell–cell communication, or from some underlying latent variable; both these mechanisms are of interest.

Recognizing the promise of spatially varying expression patterns to highlight noteworthy biology, researchers have proposed diverse methods for identifying single genes with spatially auto-correlated expression patterns, and for the related problem of identifying sets of genes that are spatially correlated with each other. Trendseek [1] uses point processes, a mainstay from the field of spatial statistics. SpatialDE [2] models spatial expression with a Gaussian process. scGCO [3] fits hidden Markov random fields. MaxSPIN [4] employs machine learning to approximate mutual information between nearby expression measurements. SpatialDM [5], which focuses on ligand-receptor pairs, uses a modified Moran’s *I* statistic. SpaGFT [6] performs a spatial Fourier transformation to analyze gene expression in the frequency domain rather than the spatial domain. Similar to the method we propose, SPARK-X [7] takes a non-parametric, kernel-based approach. Pursuing a slightly different goal, DIALOUGE [8] uses penalized estimation to infer “multi-cellular programs.” In addition, legacy methods from the field of spatial statistics, in particular Moran’s *I* [9], Geary’s *C* [10], and Lee’s *L* [11], have gained wide use.

Spatial correlation analysis has one severe limitation: most cell types are spatially organized, which induces spatial correlation among genes with cell type-specific expression. Thus spatial correlation often provides little more than an oblique readout of the cell type landscape. This pitfall can be avoided by looking one cell type at a time, but this solution misses interactions among multiple cell types. Of the above methods, only DIALOUGE models the role of cell type, but the highly structured model it fits limits the diversity of trends it can discover.

Here we introduce InSituCor, a toolkit for quickly identifying spatial correlations deserving scarce analyst attention. InSituCor identifies gene modules with spatial correlations that cannot be explained by known factors like the cell type landscape or technical effects.

## Results

**Fig. 1:**
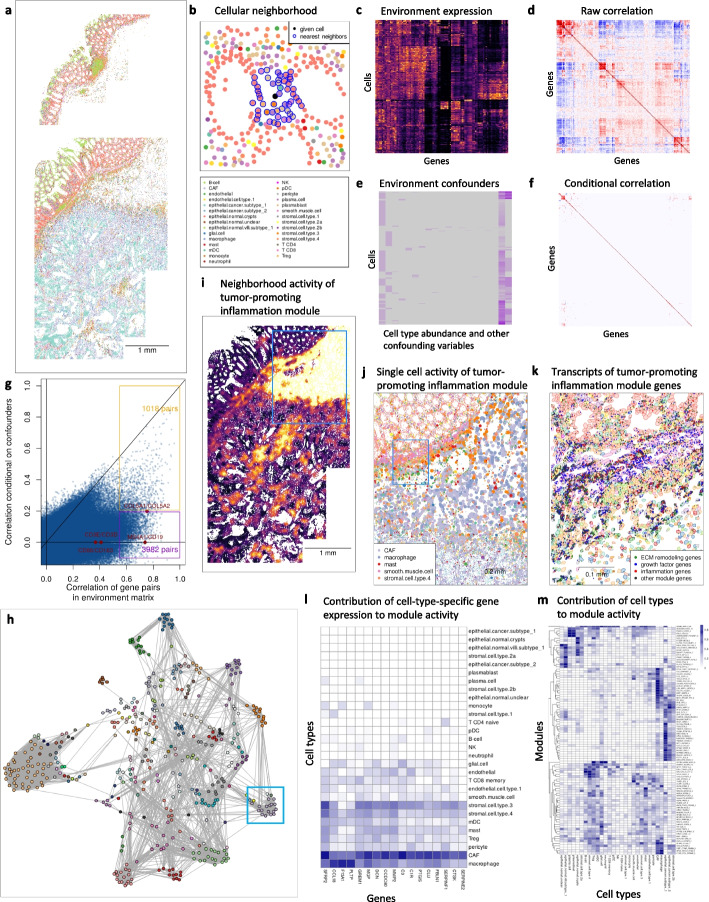
Demonstration of InSituCor workflow. **a** Cell type map of a colon cancer. Color legend applies to panels **a**, **b**, and **j**. **b** Example of a cell’s nearest neighbors, used to define its “environment expression” profile. **c** Subset of the environment expression matrix. **d** Raw correlation matrix of the environment expression matrix showing near-ubiquitous correlations (subset of 300 random genes). **e** Subset of the environment confounding matrix, encoding cell type abundance and other confounding variables in each cell’s neighborhood. **f** Correlation matrix of the environment expression matrix (**c**) conditional on the confounding matrix (**e**), over the same subset of genes as **d**. Most unconditional correlations (**d**) are fully explained by the confounding variables. **g** Raw vs. conditional correlation of environment gene expression. Selected pairs of marker genes are highlighted. **h** Network representation of the correlation between all genes in all modules. Genes with correlation > 0.2 are connected. The module explored from **i** to **k** is highlighted. **i** Environment scores for a “tumor-promoting inflammation” module. Color scale ranges from 0 expression to the 95th percentile of the module’s expression **j** Single-cell scores for the module; point size reflects cells’ module score. The window highlighted in (**i**) is shown. **k** mRNA molecules of module genes. The window highlighted in **j** is shown. **l** Estimated involvement of each cell type in each module. **m** Estimated involvement of each cell type in each gene of the highlighted module

We demonstrate InSituCor in a colon tumor profiled with a 6000-plex CosMx [12] panel (Fig. 1a). InSituCor begins by taking the expression profile of a neighborhood around each cell (Fig. 1b), building an “environment expression matrix” (Fig. 1c). Neighborhoods can be defined using K-nearest neighbors or radius-based approaches. Supplementary Figs. 1–5 detail the impact of neighborhood definition and other tuning parameters on results. Typical methods produce results akin to taking the correlation matrix of the environment expression matrix (Fig. 1d). To eliminate the influence of unwanted variables like cell type, signal strength, and background intensity, InSituCor builds an “environment confounder matrix” (Fig. 1e) summarizing these variables for each cell’s neighborhood. InSituCor defines spatial correlation as the correlation matrix of the environment expression matrix, conditional on the confounder matrix (Fig. 1f), or

1 $$\mathrm{cor}(\mathrm{environment}\;\mathrm{expression}\;\mathrm{matrix}\;\vert\;\mathrm{environment}\;\mathrm{confounding}\;\mathrm{matrix})$$

This conditional correlation matrix is the cornerstone of InSituCor analyses. It measures genes’ tendency to be expressed in the same neighborhoods, beyond what cell type and other confounders can explain. Due to the large numbers of cells analyzed, even small correlations attain strong statistical significance; for this reason, InSituCor does not compute *p*-values, as statistical significance here is a low-specificity indicator of interesting results. To speed computation, InSituCor estimates conditional correlation using a random subset of 5000 cells; larger subsets can be used for greater precision. Supplementary Fig. 2 explores the impact of subset size on accuracy.

The conditional correlation approach finds that most of the strongest spatial correlations in the unadjusted analysis do not merit analyst attention: among the top 5000 gene pairs found without adjusting for confounders, with a range of (0.62, 0.97), only 1018 had correlations > 0.2 in the conditional correlation matrix (Fig. 1g). Pairs of marker genes, for example, CD3D/CD3E and CD19/MS4A1, had strong spatial correlations but near-zero conditional correlations.

InSituCor aids comprehension by extracting modules of mutually co-expressed genes (Fig. 1h). One module discovered in this analysis consisted of 17 genes collectively suggestive of tumor-promoting inflammation (Fig. 1h, i, j, k, l). This module included genes involved in microenvironment remodeling (CCL18, MMP2, CSTK), growth factor signaling (SFRP2, GREM1, DCN, SERPINF1), and inflammation (C3, C1R, PTGIS).

Each module is scored with a weighted average of its genes; scores are calculated both for cells’ environments and for single-cell expression. A map of scores for the tumor-promoting inflammation module shows it peaking in the stroma, with smaller hotspots in the tumor bed (Fig. 1i). Looking at single-cell scores for the module, we see CAFs and macrophages driving module activity, with nearby mast cells, smooth muscle cells, and stromal cells also participating (Fig. 1j). Zooming in to resolve individual mRNAs, we see more nuanced behavior of the module genes across cell types and space (Fig. 1k).

Dozens of modules will be discovered in a typical study of ≥ 1000 genes. To help analysts prioritize, InSituCor estimates the role of each cell type in each module. Cell type involvement is summarized at the module (Fig. 1l) and at the gene level (Fig. 1m).

In a typical study, the analyst will choose confounding variables, and then derive modules with a single R command. Summary plots (Fig. 1l, m) will suggest a few modules of particular interest. The analyst can then invest real effort, carefully examining spatial plots (Fig. 1k) to develop a nuanced understanding of the modules’ behavior.

To speed up computation time, many of InSituCor’s calculations use subsets of 5000 cells. InSituCor took 2.5 min on a r5.12xlarge EC2 instance to analyze this dataset of 112,846 cells and 6000 genes.

**Fig. 2:**
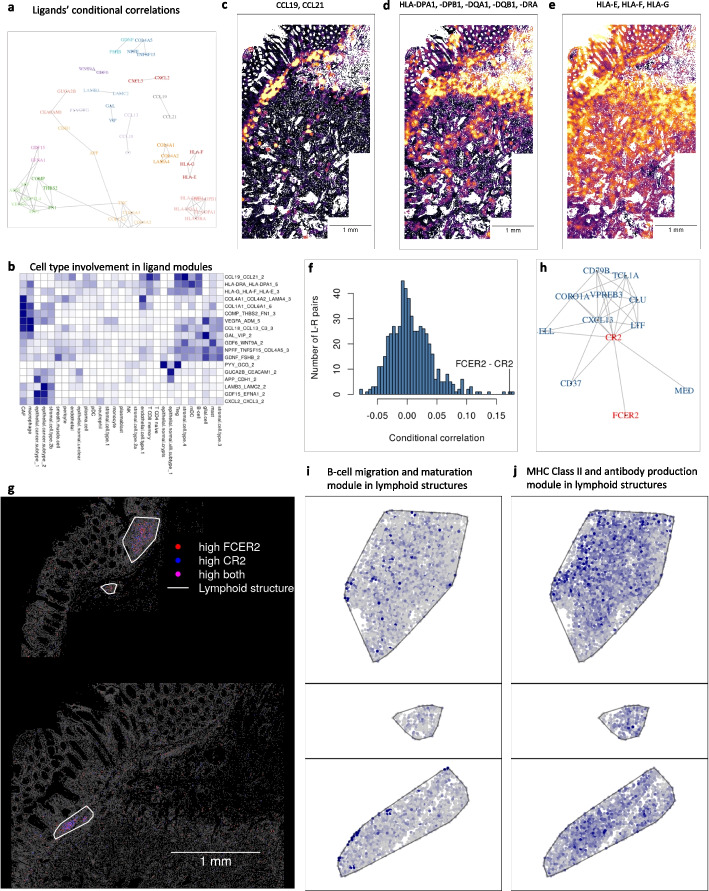
Biology-first use of InSituCor. **a** Correlation structure of 51 ligands assigned to modules. Edges show conditional correlations > 0.1, and color shows module membership. **b**. Involvement of each cell type in each module. **c**,** d**,** e** Environment expression of a modules holding chemoattractants (**c**), MHC2 antigen presentation genes (**d**), and MHC1 antigen presentation genes (**e**). Color scale ranges from 0 expression to the 95th percentile of the module’s expression. **f** Conditional correlations of 555 ligand-receptor pairs. **g** Spatial map of single-cell expression of the ligand-receptor pair FCER2 and CR2. **h** Conditional correlation network around the FCER2-CR2 ligand-receptor pair. **i** Expression of “B-cell migration and maturation” module in tertiary lymph nodes. **j** Expression of “MHC Class II antigen presentation and antibody production” module in tertiary lymph nodes

InSituCor also supports a knowledge-driven workflow: one simply examines the conditional correlation structure around genes of prior interest. To describe this tumor’s signaling environment, we re-analyzed just the dataset’s 407 ligands [13]. This produced 18 modules containing 51 ligands, many arising from multiple cell types (Fig. 2a, b). Focusing on modules involved in the anti-tumor immune response, we see a module of the chemoattractants CCL19 and CCL21 concentrated in a narrow band at the tumor periphery, a module of MHC2 antigen presentation genes diffusing slightly beyond this band, and a module of MHC1 antigen presentation genes peaking in the same region but extending further yet into the tumor bed (Fig. 2c–e). This suggests an interpretation in which a core of chemoattractant expression attracts antigen-presenting cells and an adaptive immune response radiates from this core, eliciting MHC1 expression from surrounding cells.

Ligand-receptor pairs motivate another use case, as spatial correlation suggests co-regulation, presumably by the ligand increasing the receptor’s expression or via some latent variable inducing regional expression in both genes [5]. Of the 555 ligand-receptor pairs in this panel [13], only 11 had conditional correlation > 0.1 (Fig. 2f). One correlated pair was FCER2 and CR2, both primarily expressed by B-cells. Visual examination showed their spatial variability to be driven by lymphoid structures (Fig. 2g), where B-cells had 2.57-fold (95% confidence interval 1.63–3.51) higher FCER2 and 3.43-fold (2.12–4.75) higher CR2 than B-cells elsewhere.

InSituCor can also be used to explore individual genes of high prior interest. Motivated by the above results, we examined the correlation network around FCER2 and CR2 (Fig. 2h). FCER2 had no further connections, but CR2 belonged to a densely connected network of 10 additional genes involved in B-cell development, activation, and trafficking [14–16]. This suggests the hypothesis that the genes connected to CR2 are activated downstream of FCER2–CR2 signaling.

Above, InSituCor was adapted to target genes of interest; it can also focus on spatial regions. To demonstrate this approach, we re-ran InSituCor on only the 3889 cells falling in tertiary lymphoid structures (TLS) (Supplementary Fig. 6). The results included a module capturing B-cell migration and maturation (ADAM8, ATP2A3, CEBPD, IGLC1, IGLC2, IGLL1, IGLL5, VASP) (Fig. 2i) and a module capturing MHC Class II antigen presentation and antibody production (CD74, CTSH, CTSZ, CYBA, FCRLA, HLA-DRA, HLA-DRB, IGHM, OS9, VSIR) (Fig. 2j). The TLS-only analysis returned 52 gene pairs with strong (> 0.5) conditional correlations that had been near-zero (< 0.1) in the whole-tissue analysis, suggesting that a spatially-targeted approach can discover relationships obscured in tissue-wide analyses.

**Fig. 3:**
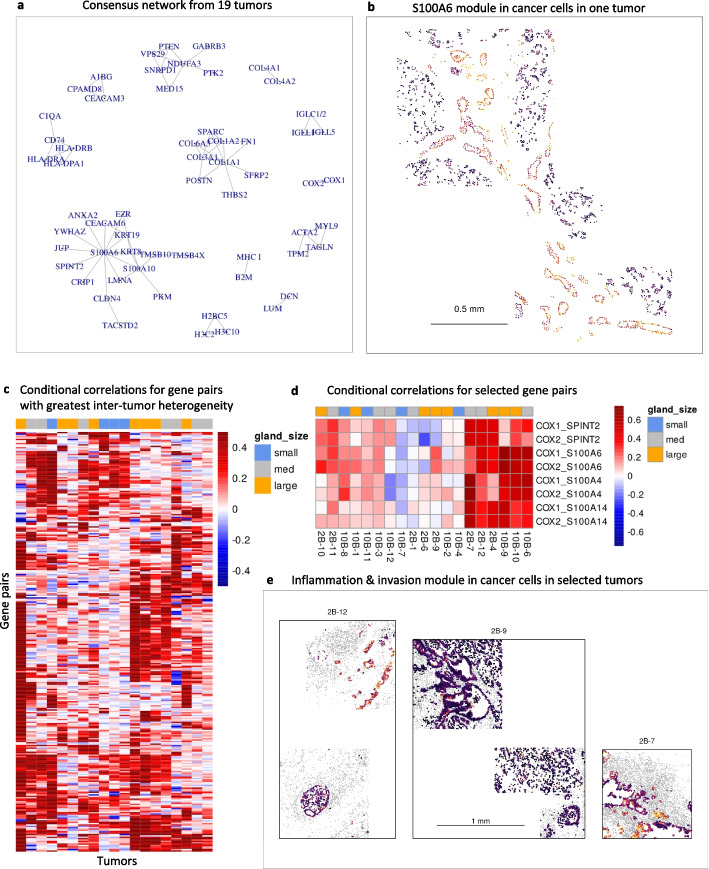
Application of InSituCor to a tissue microarray of PDAC tumors. **a** Consensus correlation structure: gene pairs with correlations > 0.3 in ≥ 17/19 tumors. **b** Expression of “S100A6” module genes (S100A6, JUP, ANXA2, EZR, CEACAM6, KRT19, KRT8, TSMB10, TSMB4X, LMNA, PKM, TACSTD2, CLDN4, CRIP1, SPRINT2) in cancer cells of tumor 10B-7. **c** Conditional correlations of 307 gene pairs showing inter-tumor heterogeneity. Each element shows a gene pair’s conditional correlation estimate from a single tumor. Top bar color denotes the gland size characterizing each tumor. **d** Tumor-specific conditional correlations from a cluster of the gene pairs from **c**. **e** Expression of a module constructed from the selected cluster of gene pairs (COX1, COX2, SPINT2, S100A4, S100A6, S100A14). Cancer cells are colored by average expression of module genes; other cells are grey

To demonstrate the use of InSituCor in multi-sample studies, we analyzed tissue microarray cores from 19 pancreatic ductal adenocarcinoma (PDAC) tumors [17]. InSituCor was run separately for each tissue, producing 19 conditional correlation matrices. We first sought conditional correlations found consistently across the tissues. 3212 gene pairs had high conditional correlations (> 0.3) in at least 17 of the 19 tissues. We used these pairs to form a consensus network of spatial correlations (Fig. 3a). This exercise returned well-known relationships, for example, a module consisting of B2M and a probe for the MHC Class I genes HLA-A, HLA-B, and HLA-C, and a module consisting of immunoglobulin lambda genes. More novel findings included a module centered around S100A6 and included genes involved in cell adhesion (CLDN4), epithelial-mesenchymal transition (S100A6, CEACAM6, TACSTD2), metabolism (PKM), and proteasome activity (TMSB10, TMBS4X). This module’s expression is driven by cancer cells and varies between tumors and spatially within tumors (Supplementary Fig. 7). In one tumor, cancer cells with high module expression form glandular structures, while cancer cells with low module expression have lost their gland structure (Fig. 3b). We hypothesize that this module captures key activities of the classical PDAC pathology that are lost in basal pathology. However, the consistent discovery of this module across the study’s tissues suggests it is variably regulated within single tumors rather than a static indicator of the cancer subtype.

Next, we sought conditional correlations with highly variable behavior across tumors. We identified 307 gene pairs that had strong conditional correlations (> 0.4) in at least 3 tumors and low conditional correlations (< 0.05) in at least 3 tumors (Fig. 3c). A module of genes for inflammation (COX1, COX2) and invasion (SPINT2, S100A4, S100A6, S100A14) shared high (> 0.4) conditional correlations in 6 tumors and attenuated or no correlation in the remaining 13 tumors (Fig. 3d). In the tumors where this gene program is active, it displayed marked spatial heterogeneity, sometimes at the level of tumor glands, and sometimes just a few cells at a time (Fig. 3e, Supplementary Fig. 8).

PDAC tumor glands form in widely varying sizes; smaller glands become more common after therapy and are dominated by basal-like cells [17]. We classified the tumors in our study by whether they were characterized by small or larger tumors or fell somewhere in between (Methods). Examining the gene pairs with high between-tumor variability, we find a module of genes displaying high conditional correlations only in tumors characterized by small-to-intermediate glands. These genes include cellular structure and adhesion (KRT7, KRT16, KRT17, KRT19, ITGA2, LAMC2, S100A10) and other functions (YWHAZ, HMGA1, MUC4). This module is largely absent from large glands and shows higher and varying expression in small glands (Supplementary Fig. 9). Another module of genes was found exclusively in tumors characterized by intermediate-to-large tumor glands. It contained genes involved in mucosal maintenance (TFF1, TFF2, AGR2), inflammation (S100P, LYZ) and others (IER3, CEACAM6). This module showed strong within-tumor variability, sometimes varying across the span of a single gland (tumor 10B-9, Supplemental Fig. 10).

## Discussion

InSituCor seeks variability conditional on the cell type landscape. If cell types are omitted from the input, for example, if a cell type is underclustered or if a rare cell type is overlooked, then the omitted cell types will give rise to modules of spatially correlated genes. A side benefit of InSituCor then is to reveal cell types missed by initial cell typing; anecdotally, we have discovered rare cell types in this way. The appropriate granularity of cell typing should be considered a design decision by the analyst. For example, if cancer cells fall in clear subclusters, a reasonable analyst might ignore this subclassification when running InSituCor, thereby obtaining a description of cancer cells based on their spatial variability rather than based on subclusters.

InSituCor names modules with their two most influential genes. Alternative approaches such as large language models or gene set enrichment analysis could also be useful. We find it practical to accept the default names for initial analysis and then carefully assign more interpretable names for modules of interest.

Most InSituCor operations are performed on data from cellular neighborhoods, with each cell’s neighborhood defined to capture the set of cells proximal enough to influence it. We prefer the K-nearest neighbors approach to radius-based neighborhoods for the theoretical reason that tissue regions packed densely with cells could be less permeable to cell–cell communications, and for the practical reason that radius-based neighborhoods in sparse tissue regions can contain so few cells that their neighborhood expression becomes statistically unstable. Undoubtedly, tissue biology is far more complex than the simple K-nearest and radius-based neighborhood concepts implemented within InSituCor: genes and cell types vary in their functional reach, and tissue structures like epithelial walls impede cell and molecule trafficking. Thus the cellular neighborhoods we measure are noisy proxies for true neighborhoods, and this noise likely attenuates InSituCor’s conditional correlation estimates. The problem of optimally defining cellular neighborhoods has not been well-studied. Should better frameworks emerge, InSituCor accepts user-defined cellular neighborhoods.

InSituCor is highly sensitive to spatially-dependent technical artifacts inducing spatial correlations, for example, field of view (FOV) effects or regions of autofluorescence or tissue peeling. Fortunately, such artifactual results are unmistakable, producing for example a gene module with outlier expression across a single FOV. Thus InSituCor serves as a secondary QC, detecting technical effects missed earlier in analysis.

Our application of InSituCor to only the tertiary lymphoid structures in the colon tumor showed that networks focused on the biology of interest can be more informative than tissue-wide networks. A straightforward extension would be to compare networks found in contrasting spatial domains, for example, tumor interior vs. tumor border. The approach for multi-sample analysis in Fig. 3 could apply directly to the domain-specific problem: InSituCor could be run separately across multiple spatial domains, and conditional correlations can be compared directly.

## Conclusions

Cell-type landscapes induce strong spatial correlation between genes, even when those genes do not vary within a cell type. In our example dataset, most of the strongest spatial correlations in the unadjusted analysis proved to be uninteresting after adjusting for cell type abundance. By conditioning on cell type and other confounders, InSituCor ignores these trends and instead reports only correlations indicating more interesting biology. It quickly—in both computational time and analyst time—isolates and summarizes spatial correlations deserving further investigation.

The InSituCor R package is available on GitHub [18].

## Methods

### InSituCor algorithm

Use *i* to index cells, *j* to index genes, and *l* to index confounder variables. Call *C(i)* the cell type of cell *i*. Call *Y* = *{Y*_*ij*_*}* the matrix of single-cell gene expression, and call *X* = *{X*_*il*_*}* the matrix of single-cell confounder values. For example, for *l* corresponding to “T-cell”, *X*_*il*_ would equal 1 for all cells with *C(i)* = “T-cell” and 0 for other cells.

InSituCor begins by defining each cell’s neighboring cells. Call *N(i)* the neighbors of cell *i*. Neighbors can be defined either with a fixed radius of as the K-nearest cells. Recognizing that both these concepts of “neighborhoods” are limited, InSituCor also accepts custom-defined cell neighborhoods, making it forward-compatible as more advanced neighborhood definitions enter the literature.

Given cells’ neighbor relationships, we then calculate each cell’s neighborhood gene expression values and neighborhood confounder values. Call these respectively ${Y}_{i,j}^{(N)}=\sum_{{i}{\prime}\in N(i)}{Y}_{{i}{\prime},j}$ and ${X}_{i,j}^{(N)}=\sum_{{i}{\prime}\in N(i)}{X}_{{i}{\prime},j}$. We then calculate InSituCor’s primary output, the correlation of the neighborhood expression matrix ${Y}_{i,j}^{(N)}$ conditional on the neighborhood confounding matrix ${X}_{i,j}^{(N)}$. To do this we first take their conditional covariance:

2 $$\text{cov}\left({\text{Y}}_{\text{i},\text{j}}^{\left(\text{N}\right)}|{\text{X}}_{\text{i},\text{j}}^{\left(\text{N}\right)}\right)=\text{cov}\left({\text{Y}}_{\text{i},\text{j}}^{\left(\text{N}\right)}\right)-\text{cov}\left({\text{Y}}_{\text{i},\text{j}}^{\left(\text{N}\right)}, {\text{X}}_{\text{i}.\text{j}}^{\left(\text{N}\right)}\right)\text{cov}{\left({\text{X}}_{\text{i},\text{j}}^{\left(\text{N}\right)}\right)}^{-1}\text{cov}\left({\text{X}}_{\text{i},\text{j}}^{\left(\text{N}\right)}, {\text{Y}}_{\text{i},\text{j}}^{\left(\text{N}\right)}\right)$$

This is equivalent to regressing the environment expression matrix on the confounders matrix and taking the covariance of the residuals. Conditional correlation is calculated by rescaling this covariance matrix to have a unit diagonal. The above formula holds for multivariate normal variables; because our environment expression matrix is produced by averaging each cell’s 50 nearest neighbors, the central limit theorem provides some assurance that multivariate normality approximately holds. Supplementary Fig. 11 explores the impact of the normality assumption.

To define modules, InSituCor creates a weighted adjacency matrix by replacing all elements of the conditional correlation matrix below a threshold (default = 0.2) with 0. It then clusters this graph using the Leiden algorithm [19]. Modules larger than a user-specified threshold are sent through Leiden clustering a second time with double the initial resolution parameter. Modules smaller than a user-specified threshold are thrown out, as are modules whose average conditional correlation falls below a user-specified threshold.

Module scores are calculated as weighted averages of their genes: for module *m* with weights *w*_*j*_, the module’s score for cell *i* is calculated ${M}_{i,m}={\sum }_{j }{w}_{j}{Y}_{ij}$, and the score for cell *i*’s neighborhood is ${M}_{i,m}^{(N)}={\sum }_{j }{w}_{j}{Y}_{i,j}^{(N)}$. To mitigate the heteroscedasticity found in genes of varying expression levels, InSituCor’s default uses inverse square root weighting to account for the Poisson-like mean–variance relation seen in count data, defining *w*_*j*_ = mean( ${Y}_{\bullet ,j}^{(N)}$)^−0.5^.

Cell type attribution scores are computed in two steps. First, we compute the contribution of each of the module’s genes in each cell type to the module. We summarize this with the correlation between two quantities: cells’ environment scores for the module, and cells’ neighborhood expression of the gene in question by the cell type in question. That is, for module *k*, we report A(*j,c*) = cor(M^(N)^_.k_, Y^(N,c)^_.∙,j_), where *Y*^*(N,c)*^_*.i,j*_ is the total expression of gene *j* in cells of cell type *c* in the neighborhood of cell *i*. Second, we summarize the role of a cell type in a module, considering all genes, as *A(c)* = *max*_*j*_*{A(j,c)}*. This produces high attribution scores for cell types that contribute heavily to any of a module’s genes.

### Analysis of CosMx colon cancer dataset

A 13 mm × 12 mm × 5 μm section of colorectal adenocarcinoma was profiled using the standard CosMx RNA protocol and a 6000-plex, pre-commercial version of the CosMx 6 K Discovery RNA panel. Seventy-three fields of view were placed according to markup of a serial hematoxylin and eosin (H&E) stain, focusing on normal intestinal mucosa, lymphoid aggregates, and cancer. The slide was imaged with a 5-channel morphology panel (PanCK, CD68, CD298/B2M, CD45, DAPI). Analysis began with the counts matrix and cell metadata flat files exported by AtoMx. Single-cell expression profiles were normalized by dividing each cell’s expression profile by its total counts. Cell types were defined using the InSituType [20] R package’s semi-supervised mode to fit de novo clusters while also finding known cell types based on reference profiles of immune cells [21] and of mesenchymal cells [22].

InSituCor was run on the normalized count data with a subset size (“max_cells” argument) of 10,000. It conditioned on neighborhood cell type proportions, mean neighborhood total counts per cell, and mean neighborhood negative control probe counts per cell. Modules with > 20 genes were subclustered, and modules with < 3 genes were discarded. The “raw” spatial correlation matrix was calculated with cor(Y^(N)^).

Ligands and ligand-receptor pairs were taken from CellChatDB [5]. Tertiary lymphoid structures were defined by clustering B-cell locations with dbscan [23], with a radius = 0.02 mm. The 3 largest clusters (130, 501, and 905 B-cells) were called tertiary lymphoid structures; the next-largest cluster had just 28 B-cells. Differential expression analysis of B-cells in vs. out of TLS’s used a *t*-test on linear-scale normalized counts; the delta method was used to derive confidence intervals for the ratios of gene expression between these groups.

### Analysis of CosMx PDAC dataset

Three TMA cores with < 3000 cells were removed as having insufficient sample size to power InSituCor. Cells with < 20 counts were removed. Cell typing was performed as in (18), classifying cells as malignant based on PanCK immunofluorescence stain, then using InSituType’s supervised mode to assign the remaining cells to reference pancreatic, immune, and stroma cell types. Single-cell expression profiles were normalized by total counts. InSituCor was run using normalized count data, a radius of 0.05 mm, conditioning on cell type, FOV ID, cells’ total RNA counts, and total negative control probe counts. “Consensus” conditional correlations were defined as all gene pairs with conditional correlation > 0.3 in at least 17/19 tumors. Gene pairs with high inter-tumor heterogeneity were defined as those with conditional correlation > 0.3 in at least 3/19 tumors and conditional correlation < 0.05 in at least 3/19 tumors. Module scores were calculated as cells’ average normalized expression of module genes. To classify tumors as dominated by large vs. small glands, we clustered the spatial positions of malignant cells into glands using DBSCAN, then split individual glands into either small or large classes based on the number of cells they contain. Cells not assigned to any cluster are left as NA and filtered out for the downstream analysis. To define the boundary between small and large glands, we fit a two-component Gaussian mixture model to gland sizes and found the intersection of the small and large gland peaks, resulting in a cutoff of 46 cells per gland. Tumor cores were then split into three equally sized categories based on their proportion of malignant cells in each gland type.

## Supplementary Information

Additional file 1: Contains all supplementary figures and analyses referenced in this article, including analyses guardbanding InSituCor’s tuning parameters (Fig2 S1-5), additional results from InSituCor run on tertiary lymphoid structures within the colon dataset (Fig S6), results from the PDAC analysis (Fig S7-10), and an evaluation of the normality assumption (Fig S11).

Additional file 2: Review History.

## Data Availability

The colorectal cancer CosMx dataset [24] used during the current study is available on Figshare. The PDAC dataset is on Figshare in two versions. The first version [25] is a complete dataset. This version is embargoed until 2026 to preserve the novelty of the manuscript it was generated for. The second version [26] is identical to the first, but obscures all gene names not mentioned in this work. The code used in data analysis [27] is available at https://zenodo.org/records/14751985_ under an MIT license.
