# Supplementary material for: InSituCor: exploring spatially correlated genes conditional on the cell type landscape
Source: Genome Biol. 2025 Apr 24;26:105. doi: 10.1186/s13059-025-03554-1 (PMC12020328; doi:10.1186/s13059-025-03554-1)
Supplement: Supplementary file 1 — Additional file 1: Contains all supplementary figures and analyses referenced in this article, including analyses guardbanding InSituCor’s tuning parameters (Fig2 S1-5), additional results from InSituCor run on tertiary lymphoid structures within the colon dataset (Fig S6), results from the PDAC analysis (Fig S7-10), and an evaluation of the normality assumption (Fig S11). [file 13059_2025_3554_MOESM1_ESM.docx]

**Supplementary Material**

**Impact of tuning parameters on results**

To explore the impact of tuning parameters InSituCor’s results, we re-analyzed the colon cancer dataset under a range of settings. First, we defined cellular neighborhoods using increasing “K” argument values (number of nearest neighbors) and increasing “radius” argument values (Supplementary Figure 2). The k-nearest and radius-based approaches returned broadly consistent results, especially among positive correlations, which we are more interested in. Larger neighborhoods uncovered more correlations.

Second, we varied the “max_cells” parameter, which determines the size of the subset of cells from which the conditional correlation matrix is derived. To speed calculations, we analyzed 50 genes (1225 gene pairs). We ran InSituCor once using all cells, and treated this result as truth. Then, for each of a range of max_cells choices, we ran InSituCor 10 times, and for each gene pair we recorded the root mean square error (rMSE) between the truth and the subset results. The default value of 5000 cells produced an average rMSE of 0.014, and in the 10 runs using 5000-cell subsets, only 0.57% of gene pairs fell on a different side of the default correlation threshold (0.1) from the truth.

Finally, we varied the “corthresh” argument, which controls the threshold for including conditional correlations in the adjacency matrix input to Leiden clustering of genes into modules. We compared results from a very low value of 0.025 to results from higher values. Applying a more stringent threshold for conditional correlations led to fewer, smaller gene modules (Supplementary Figures 4-6). As the threshold was set increasingly stringently, the content of modules changed little apart from loss and/or splitting of weaker modules.


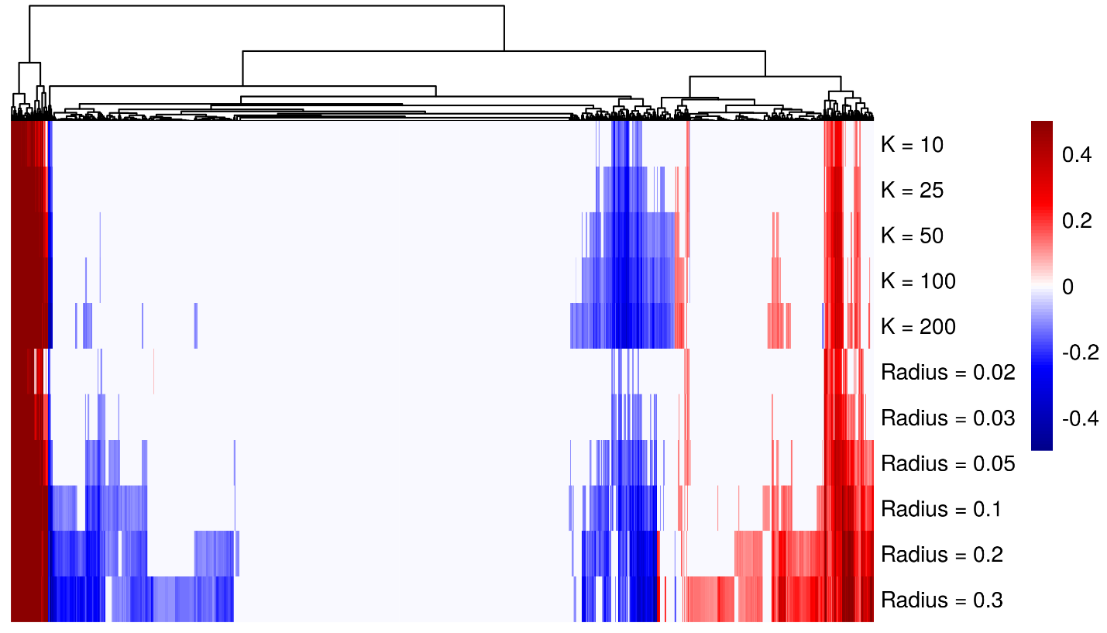


**Fig S1: impact of neighborhood definition on conditional correlation estimates.** Each row shows a neighborhood definition; each column shows conditional correlation estimates for a single gene pair. Results from 50 randomly selected genes (1225 pairs) are shown. Conditional correlations falling in (-0.1, 0.1) are set to zero to focus on results in the reportable range.


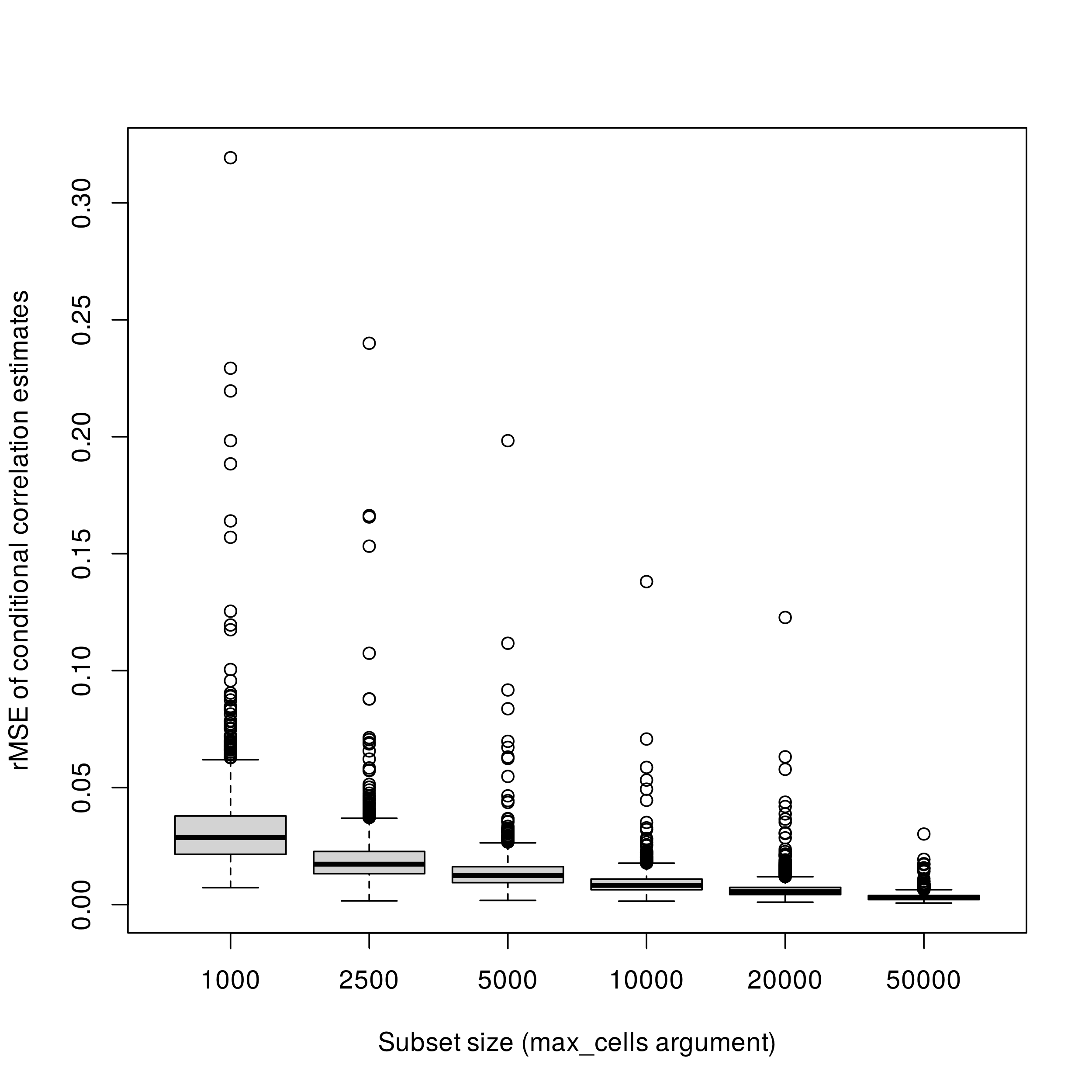


**Fig S2: impact of subset size on conditional correlation estimates.** For 50 genes (1225 gene pairs), a ground truth of conditional correlations was derived by running InSituCor across the entire colon cancer dataset. Then InSituCor was run 10 times for each of a range of subset sizes. For each gene pair’s conditional correlation, we recorded the rMSE of the subset-derived estimates vs. the ground truth.


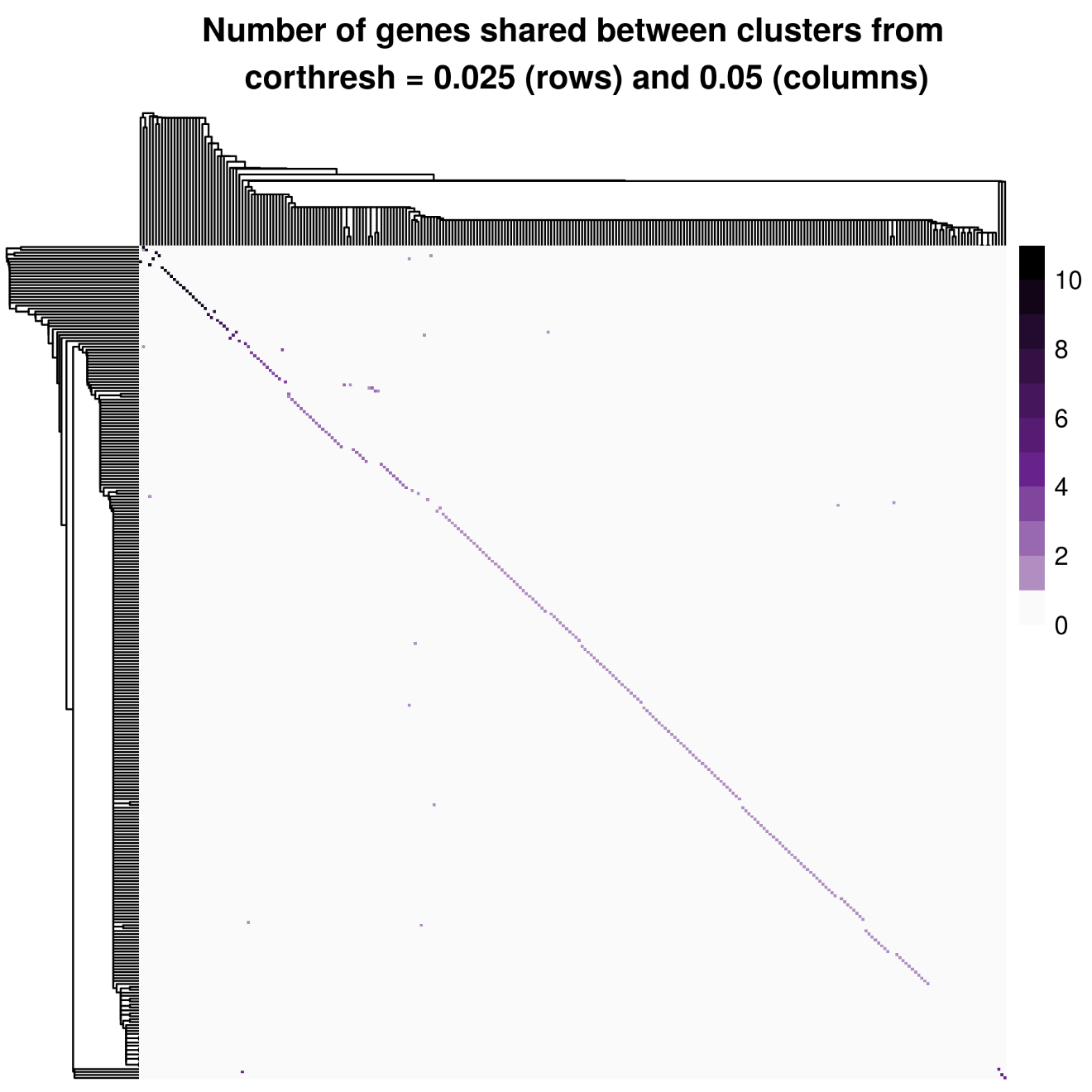


**Fig S3: overlap in modules’ gene content when using corthresh = 0.025 and 0.05.** Rows are gene modules from corthresh = 0.025; columns are gene modules from corthresh = 0.05. Cell color denotes the number of genes shared between modules. The color scale saturates at 10 shared genes; some modules share more than this.


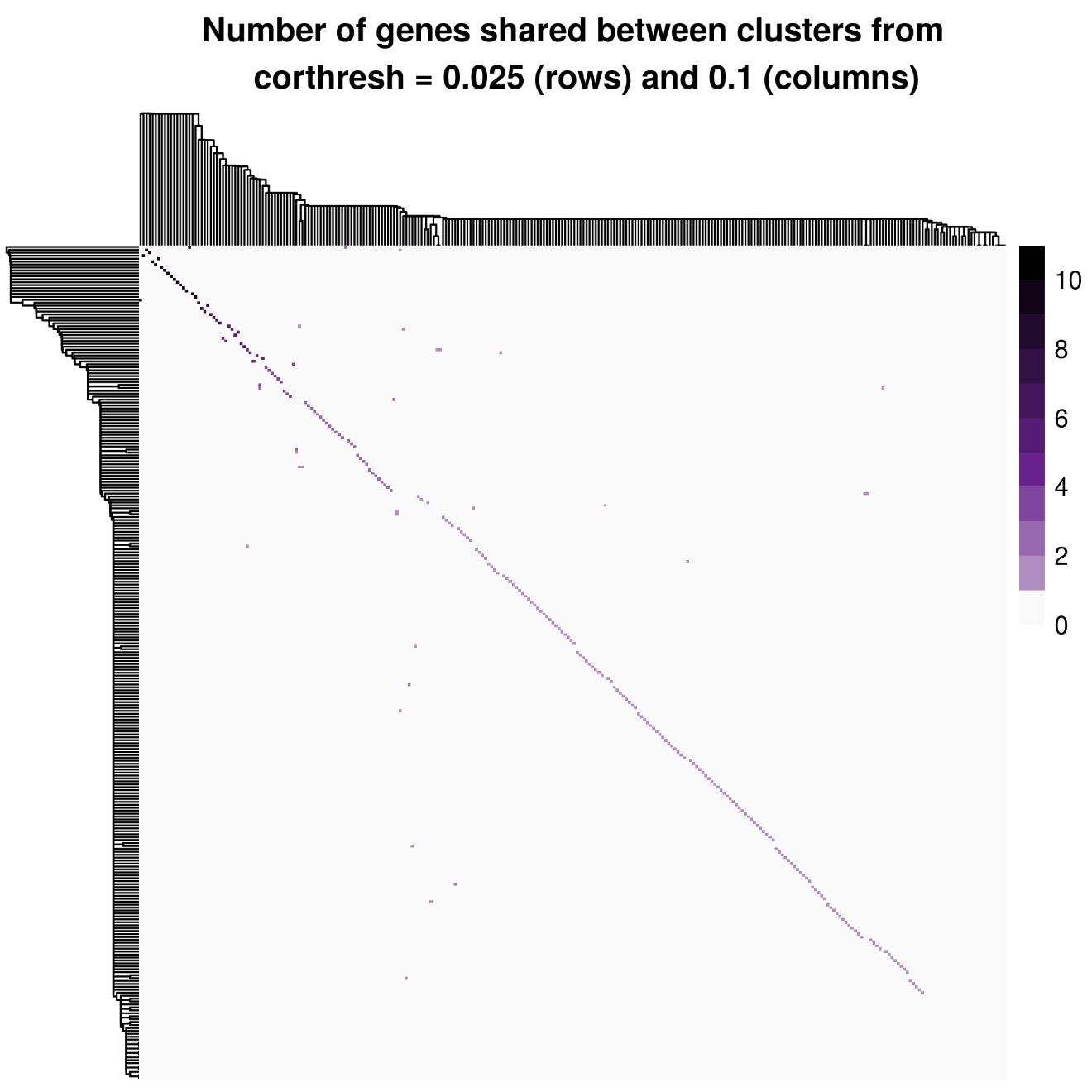


**Fig S4: overlap in modules’ gene content when using corthresh = 0.025 and 0.1.** Rows are gene modules from corthresh = 0.025; columns are gene modules from corthresh = 0.1. Cell color denotes the number of genes shared between modules. The color scale saturates at 10 shared genes; some modules share more than this.


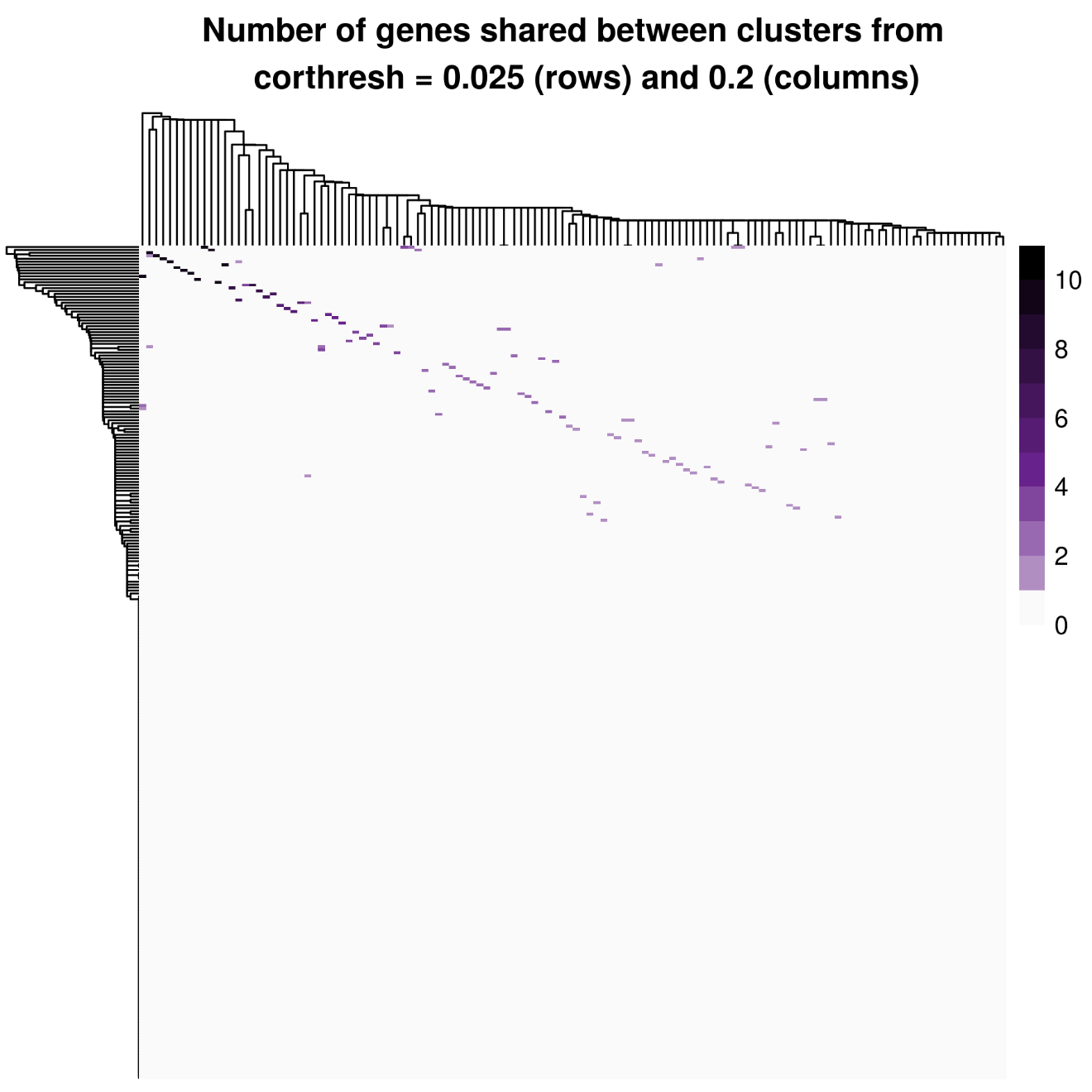


**Fig S5: overlap in modules’ gene content when using corthresh = 0.025 and 0.2.** Rows are gene modules from corthresh = 0.025; columns are gene modules from corthresh = 0.2. Cell color denotes the number of genes shared between modules. The color scale saturates at 10 shared genes; some modules share more than this.

**
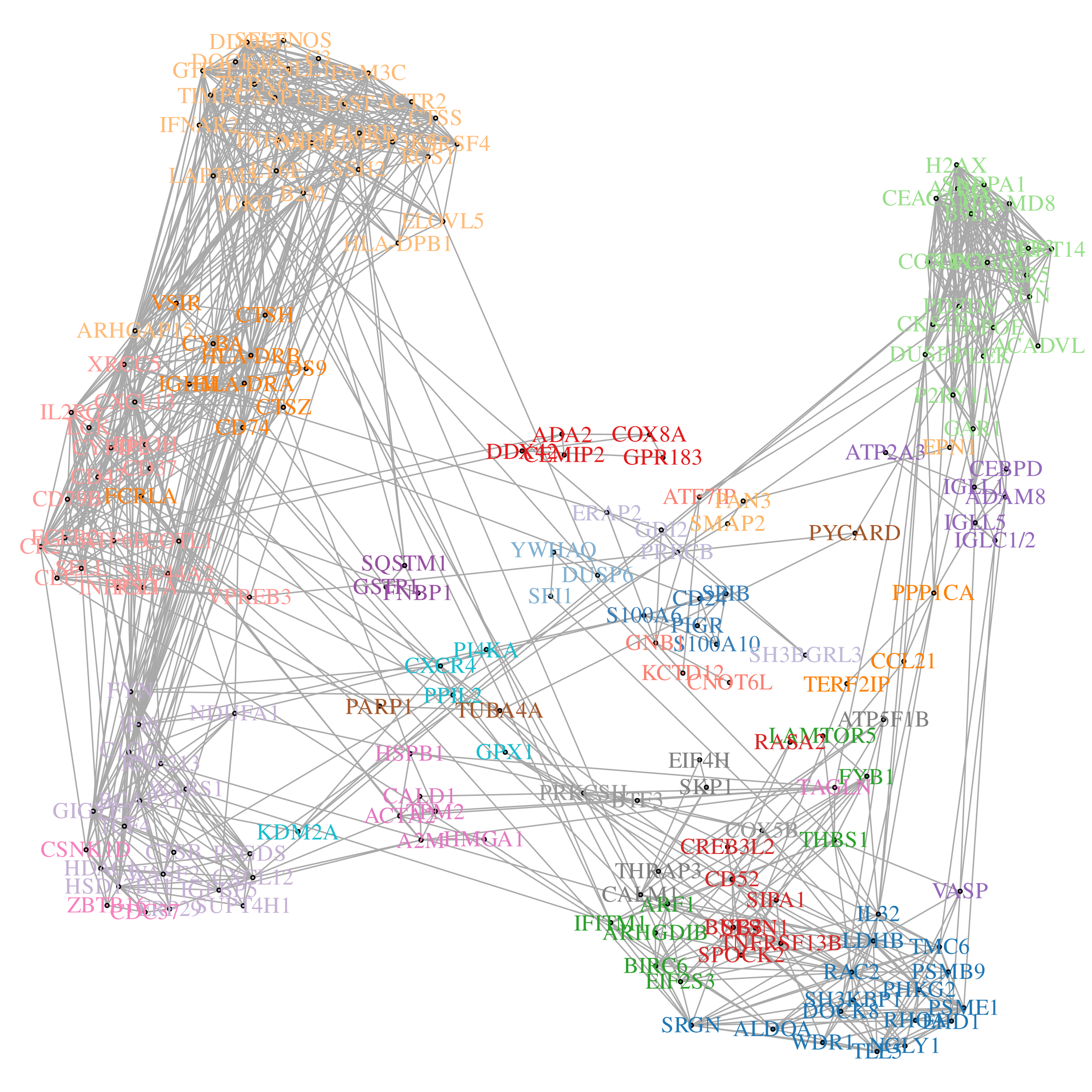
**

**Fig S6: conditional correlation network from InSituCor run on tertiary lymphoid structures within colon tissue.** Edges connect genes with conditional correlation > 0.3. Color denotes module memberhip.

**
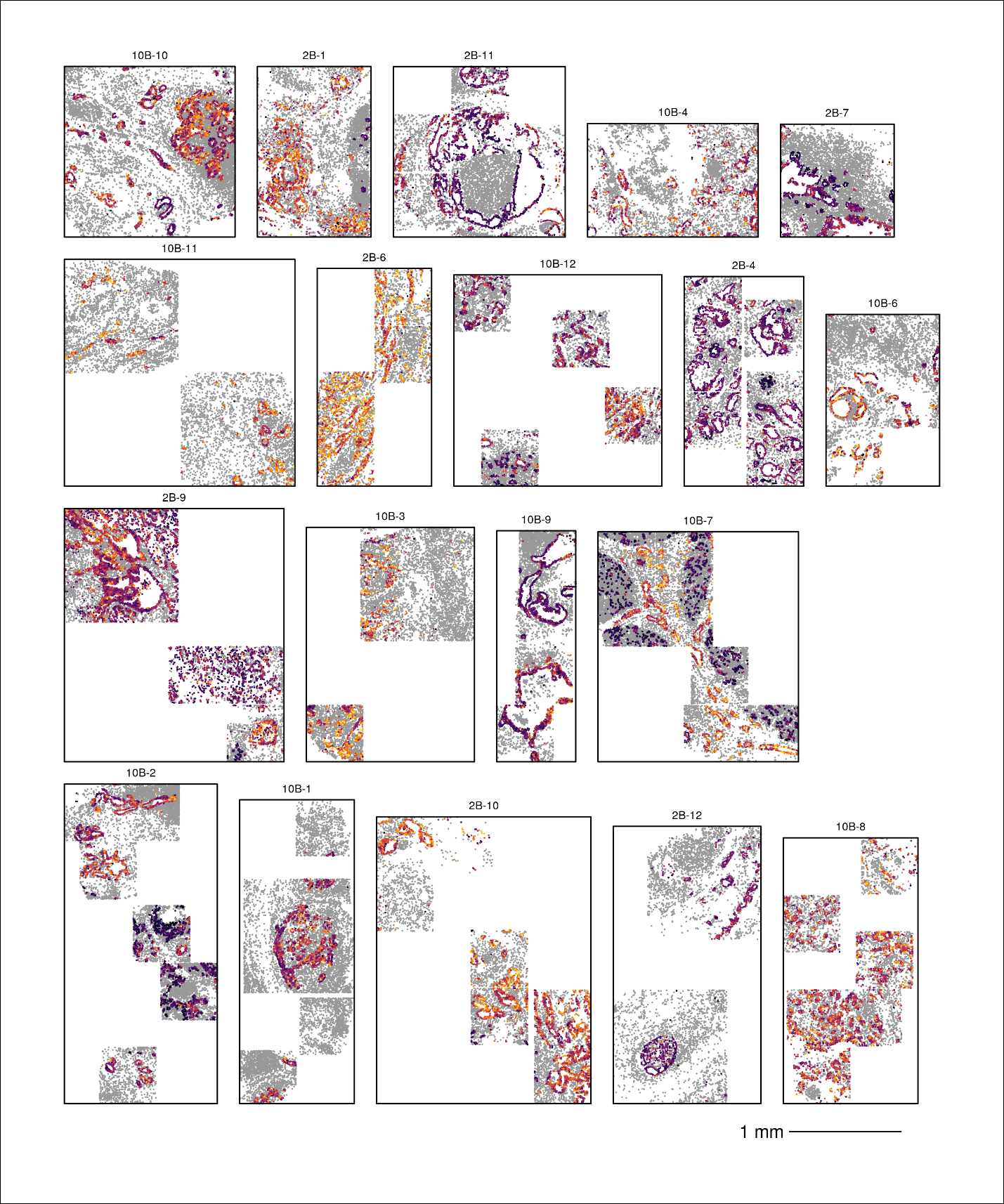
**

**Fig S7: Expression of S100A6 module in PDAC tumors.** Malignant cells are colored by their average expression of the module genes S100A6, JUP, ANXA2, EZR, CEACAM6, KRT19, KRT8, TMSB10, TMSB4X, LMNA, PKM, TACSTD2, CLDN4, CRIP1, and SPINT2. Color scale ranges from no module expression (black) to 99^th^ percentile module expression (yellow). Other cell types are grey.

**
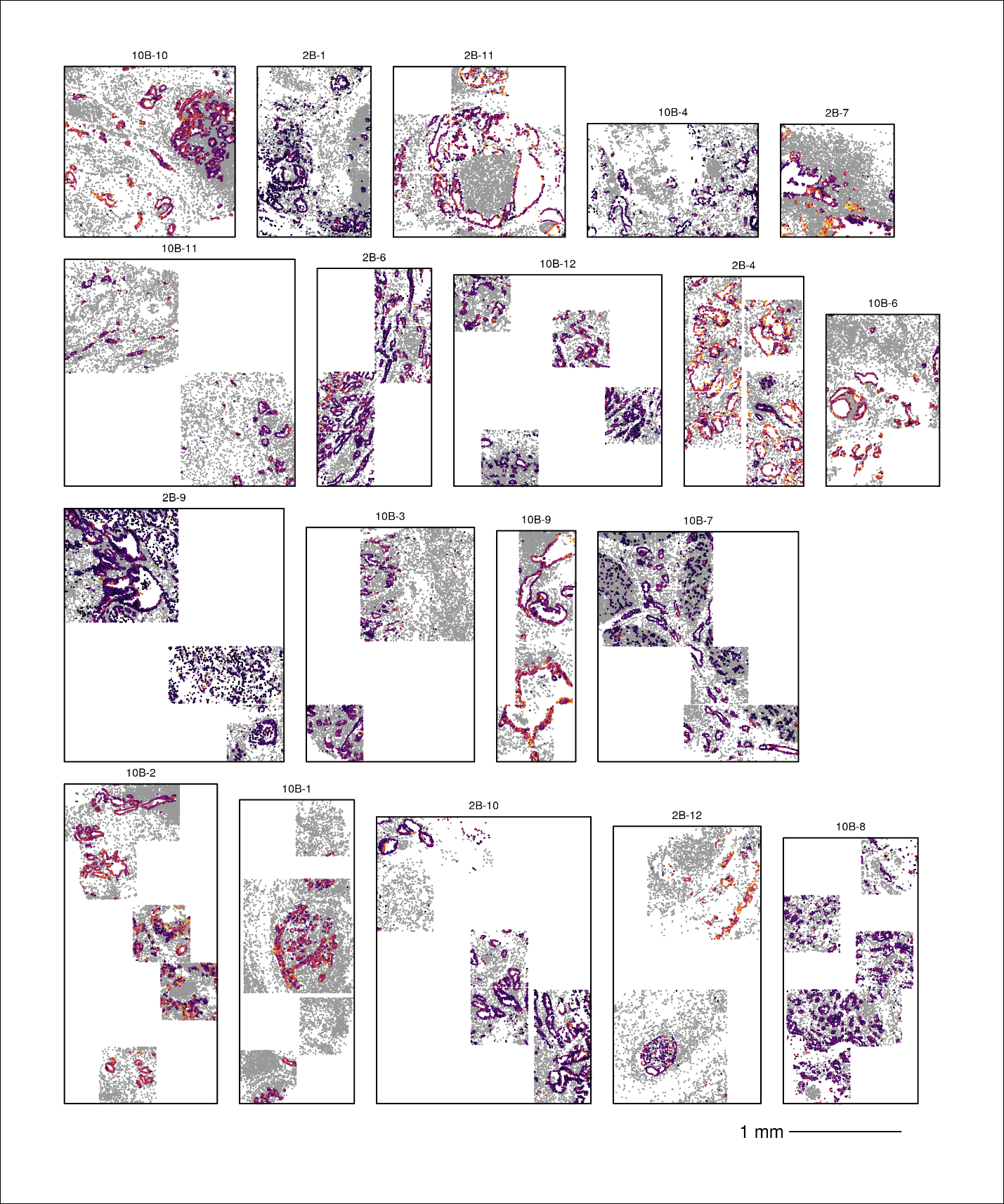
**

**Fig S8: Expression inflammation and invasion module in PDAC tumors.** Malignant cells are colored by their average expression of the module genes COX1, COX2, SPINT2, S100A4, S100A6, and S100A14. Color scale ranges from no module expression (black) to 99^th^ percentile module expression (yellow). Other cell types are grey.

**
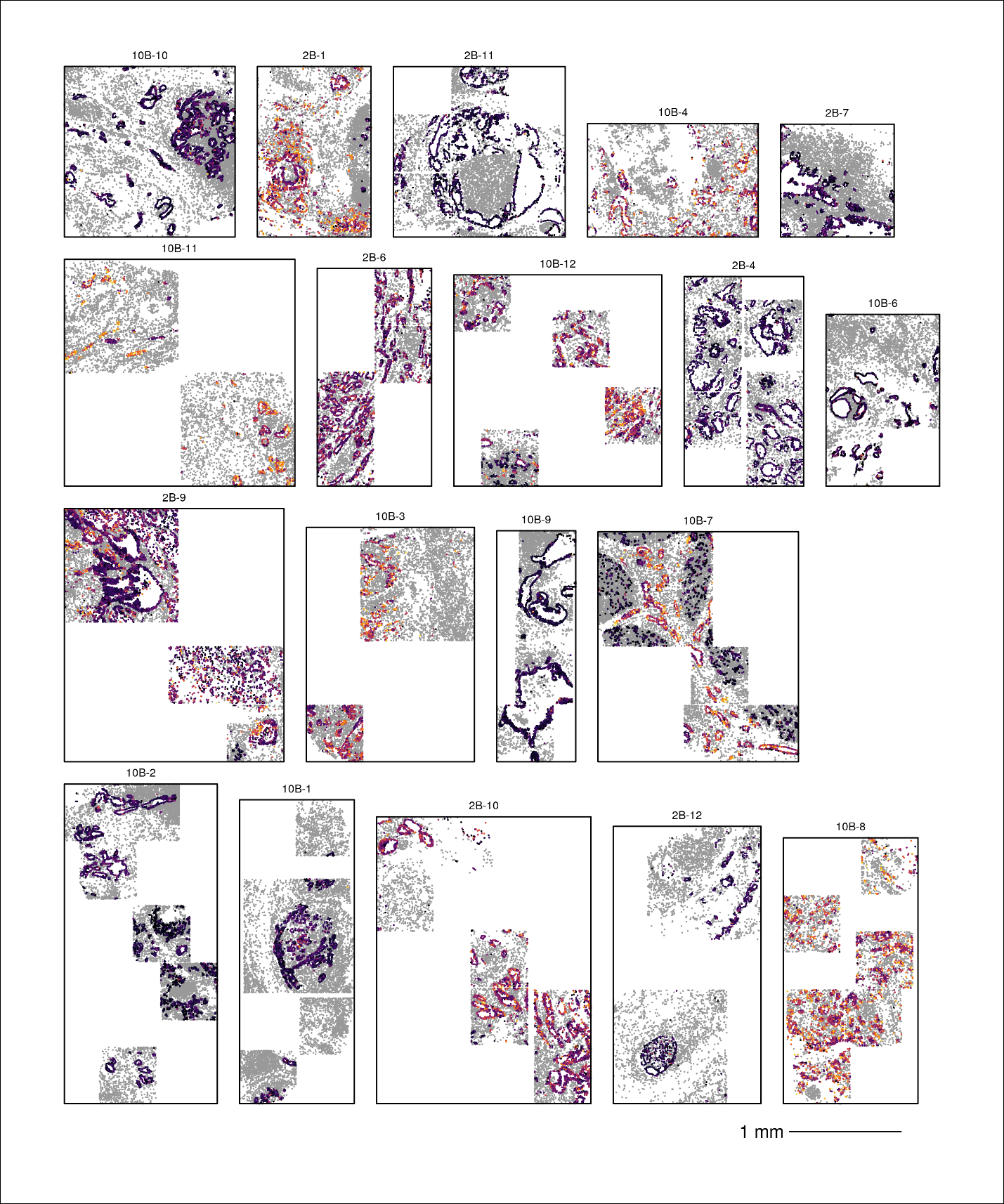
**

**Fig S9: Expression of module found in only tumors dominated by small glands.** Malignant cells are colored by their average expression of the module genes KRT7, KRT16, KRT17, KRT19, ITGA2, LAMC2, S100A10, YWHAZ, HMGA1, and MUC4. Color scale ranges from no module expression (black) to 99^th^ percentile module expression (yellow). Other cell types are grey.


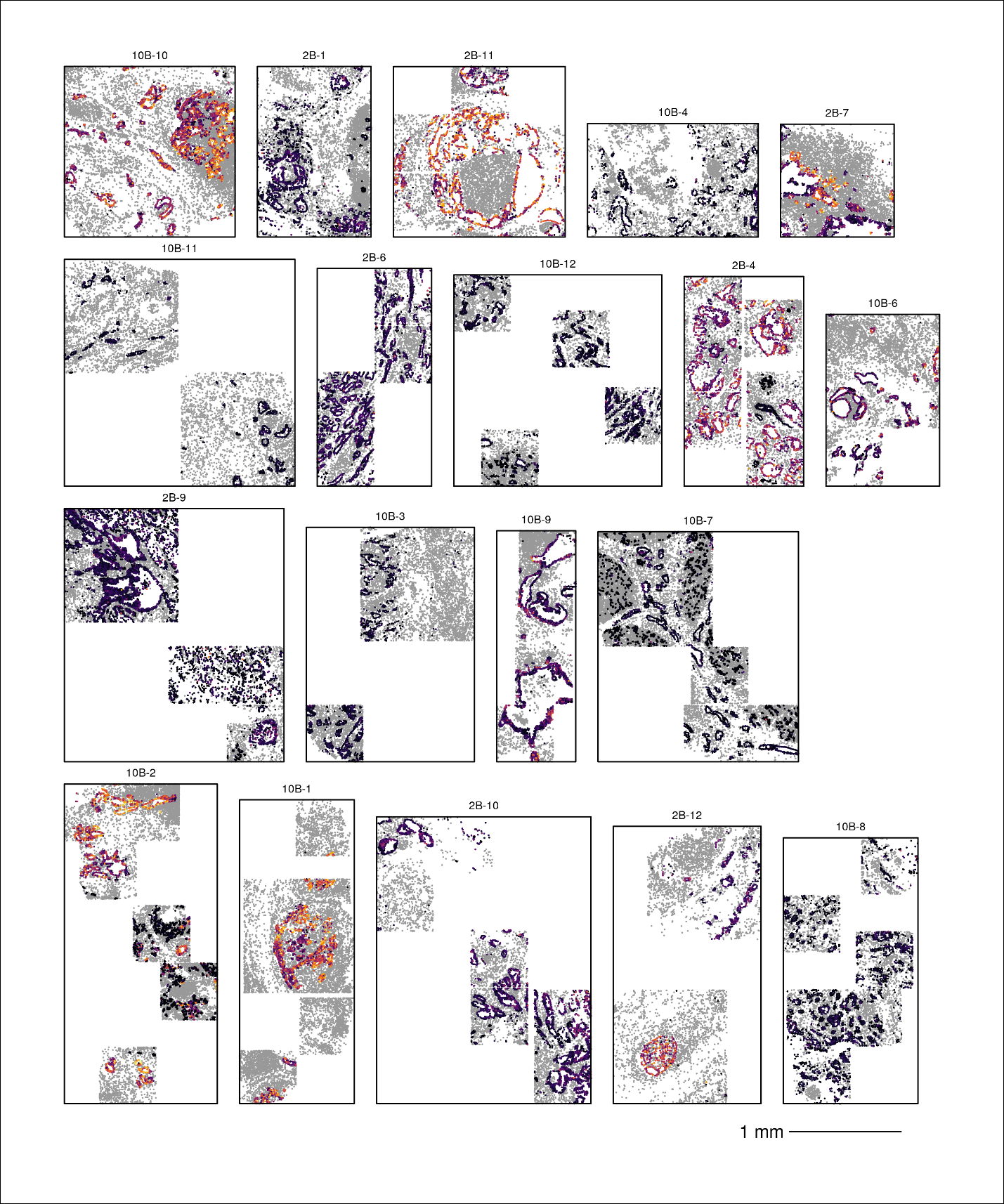


**Fig S10: Expression of module found in tumors dominated by large glands.** Malignant cells are colored by their average expression of the module TFF1, TFF2, S100P, AGR2, IER3, LYZ, and CEACAM6. Color scale ranges from no module expression (black) to 99^th^ percentile module expression (yellow). Other cell types are grey.

**Evaluation of normality assumption**

InSituCor’s calculation of conditional correlation relies on the assumption that the neighborhood expression matrix has Gaussian noise. Although single gene expression values are not Gaussian, our normality assumption is not unreasonable. Because InSituCor analyzes cellular neighborhoods, taking the average expression over a given cell’s ~50 nearest neighbors, the central limit theorem tells us that these averages converge to normality as sample size increases.

To test the reasonableness of this normality assumption, we implemented an alternative approach based on gamma regression. Specifically, for each pair of genes, we used a Generalized Linear Model with a gamma family and a log link to predict the neighborhood expression of gene1 on the neighborhood expression of gene2 and the neighborhood confounders matrix. We then saved the partial correlation between gene1 and gene2.

Compared to InSituCor, getting partial correlations from gamma regression was slow: while InSituCor took 0.56 seconds to analyze a set of 100 genes, gamma regressions took 736.22 seconds, 1315-fold longer. Since complexity rises with the square of the number of genes, we project that this method would take just under 30 days to compute across the entire CosMx 6000plex panel.

Reassuringly, our limited set of gamma regressions returned results highly concordant with InSituCor (Supplementary Figure 1).


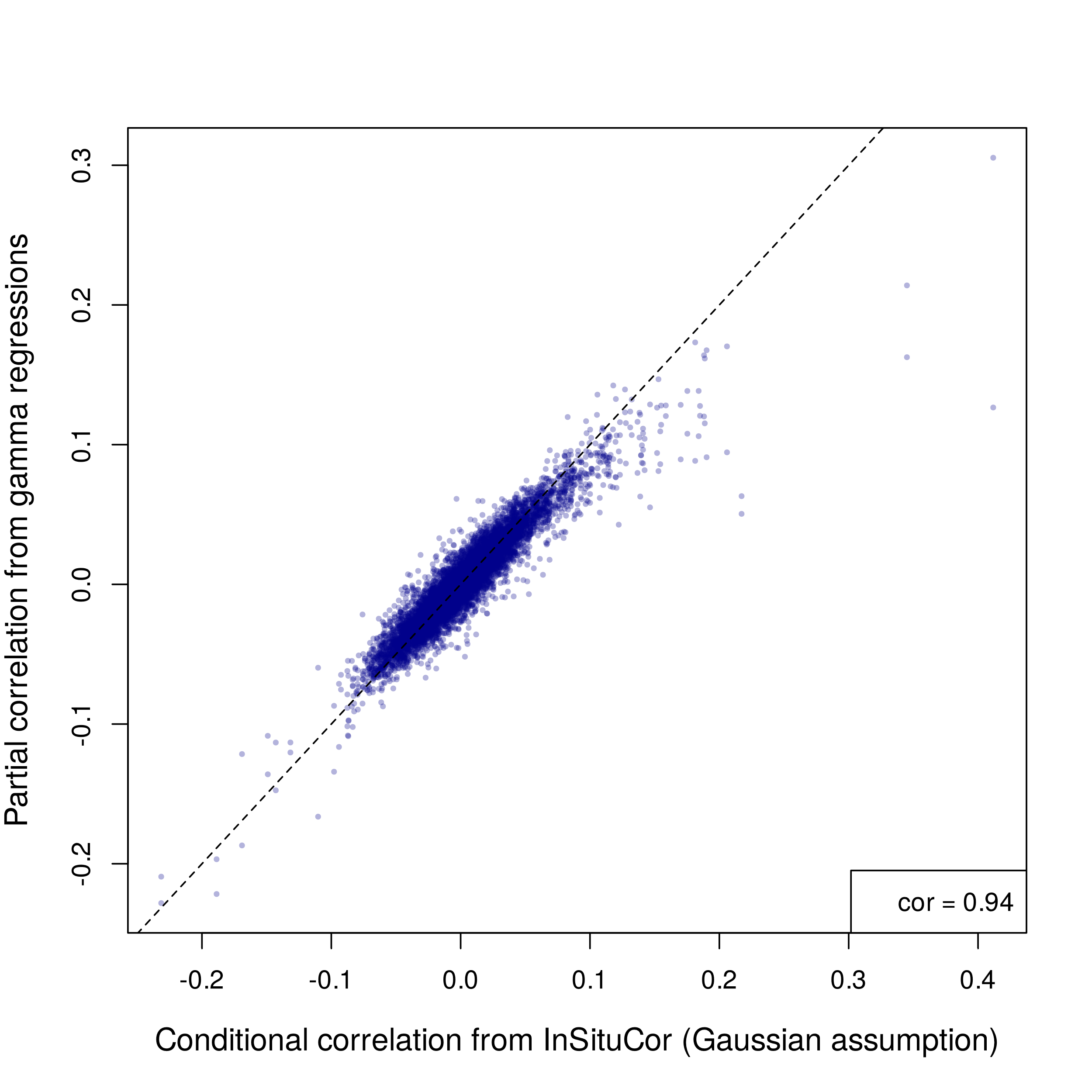


**Fig S11: concordance between InSituCor and equivalent approach using gamma regression.** For 100 genes, pairwise conditional correlations from InSituCor and partial correlations from gamma regression on neighborhood gene expression.
