# Supplementary material for: InSituCor: exploring spatially correlated genes conditional on the cell type landscape
Source: Genome Biol. 2025 Apr 24;26:105. doi: 10.1186/s13059-025-03554-1 (PMC12020328; doi:10.1186/s13059-025-03554-1)
Supplement: Supplementary file 2 — Additional file 2: Review History. [file 13059_2025_3554_MOESM2_ESM.docx]

**1^st^ round**

**Reviewer 1**

The authors propose an innovative solution, InSituCor, to study gene coregulation by regressing out the predominant source of spatial variation, cell types. The flexibility of the approach allows additional covariates to be regressed out as needed. Using the proposed approach, the authors show how phenotypes associated with tumour promotion are detected de novo, highlight the potential of the method in discovering novel molecular mechanisms. The growing plex of spatial technologies will require such tools to enable exploration of molecular phenotypes beyond cell types. Despite the potential of this method, I feel the comments below need to be addressed to ensure users are more informed on how best to apply the method. My main concern is with the lack of details in the methodology reported. I also feel the assumption of normality is unwarranted as the distribution of the data can be modelled using existing statistical tools. The authors need to address this or provide ample evidence supporting the validity of the assumption. After my comments are addressed, the proposed methods will empower spatial biologists to hone in on subtle molecular phenotypes.

Major comments:
1. The methods section is lacking in detail.
a. Though the methodological detail of InSituCor is provided to a certain extent, details regarding the preprocessing of the data (QC, normalisation, cell typing, …) are missing. Without these, readers are not likely to be able to reproduce the results in this manuscript, nor figure out how they should preprocess their own data to best work with InSituCor.
b. The comprehensive list of variables regressed out and how these are computed also needs to be clarified in the methods. Specifically, I would want to know how the background intensity (library size) is defined as these affect the covariances. The field of view (FOV) is known to be a batch effect in InSitu spatial technologies. It would be good to know whether this effect is present and whether it has been regressed out as well.
c. The authors also mention that the module score is computed using a weighted average of genes in the module. The weights used should be specified more explicitly (I assume these are somehow based on the correlation strengths).
d. The definition of cell type attribution scores is unclear to me. It would be easier to understand how this is computed if it is defined clearly using mathematical notation.
2. The authors assumed normality and suggested that averaging out the expression of 50 cells will result in normality due to the central limit theorem. However, if the counts of genes within these individual cells is relatively low (which is the case with most in situ technologies), averaged counts will also be very low and will therefore start resembling a Gamma distribution rather than a normal distribution. In such a setting, instead of simplifying the correlation estimate through a normality assumption, it would be better to fit a generalised linear model with a Poisson (if summing counts), or Gamma family (as means will still have to be positive), and then compute covariances. If the authors still believe that a normal approximation is valid, there needs to provide additional data (possibly in the form of supplementary figures), supporting this claim or showing to what extent normality is true (i.e., is it only valid for highly expressed genes?).
3. Neighbourhoods for the spatial correlation are defined as the 50 nearest cells to each cell (this should be mentioned in the results section as well). Neighbourhoods based on nearest neighbours will differ in size for dense and sparse regions. The authors should discuss the implications of neighbourhoods defined as such in their discussion section (i.e., will this mean networks are more likely to represent sparse or dense regions?). This should be studied, and guidelines provided to users on what to expect if working with tissues where the cell density varies greatly. Theoretically, spatial correlations are subject to the distances (radius around cells) and could degrade beyond certain distances (this is the basis behind cross-type spatial functions such as the L-cross function). Alternatively, neighbourhoods could be defined by using a fixed radius (or contributions could be weighted softly using a Gaussian function, however, this could complicate the resultant statistics).
4. Gene regulation is context specific with the context potentially being a spatial domain. We would expect different regulatory networks to be active in tumour regions compared to surrounding normal tissue. The proposed approach will mask correlations that are present only in a small region of the tissue. An extreme example of this would be when genes are co-expressed in a small region, but randomly expressed else where (or either one of the genes is primarily expressed elsewhere). As correlation is measured globally, these local associations would be missed. It will enhance the method if the authors allow users to define domains and run the analysis within domains to obtain domain-specific networks. This can be addressed by simply explaining in text this possibility and emphasising that users should then perform analysis within each domain. Alternatively, the method can be designed to take domain as an input and produce domain-specific networks (while potentially highlighting edges that are common across domains).
5. The authors sub-sample the data (5000 cells) to estimate the correlations. Given that the sampling rate is very low (~4%), the authors need to provide supplementary results to demonstrate the stability of estimates across repeated random samplings at this rate and at higher rates. It is important for users to know how the effect of sampling rates vary and what they should expect as they trade off accuracy for computing times. I suspect the estimates would be reliable but it will be good to see evidence of this.

Minor comments:
1. Panels c-f and l-m in figure 1 need axis titles to make it clear what is being plot.
2. Legends for the heatmap and spatial plots (1l, 2c-e) are missing in both figures. It is important to know the scales of these values and whether they have been rescaled for visualisation purposes.
3. It would help to have heading for some panels to make it easier to read the figures on their own. For instance, 1i could have the heading (or legend title) "Tumour-promoting inflammation score", that makes it easy to infer what is plot. Likewise, for the heatmaps.
4. The first sentence in the abstract is too open-ended and can be trimmed out to emphasise on the problem that InSituCor is designed to address.
5. By formally defining two matrices, the environment matrix (say Y), and the covariate/confounder matrix (X), equation 1 and 2 can be combined to provide a more concise definition.
6. The correlation analysis performed by InSituCor can possibly shed light on regulatory associations. I am curious to see how many of the nodes are known human transcription factors and where these are the central nodes in the network obtained by InSituCor. Besides a module-based analysis, central nodes (with higher degree centrality) could be an independent means to identify regions in the tissue where a known transcription factor is active. If the authors identify any such genes, it would be a strong demonstration to an alternative approach to interpret biology using InSituCor.
7. Rather than manually inferring tumour promoting inflammation from the 17 genes in the modules, the authors could use gene-set enrichment analysis (with the 6000 genes in the panel as background) to identify common processes represented by their module using existing tools such as vissE (PMID: 38331751) or EnrichmentMap (PMID: 30664679).
8. R-based bioinformatics tools are often more widely adopted when submitted to Bioconductor (or CRAN). I would advise the authors to attempt this submission to improve dissemination. It also ensures that the package works seamlessly with other Bioconductor packages. Detailed instructions can be found at https://contributions.bioconductor.org/bioconductor-package-submissions.html. Bioconductor staff are very helpful and will guide on how compliance can be achieved during peer-review.

**Reviewer 2**

Danaher et al. created a pipeline to look at spatial gene correlations while removing variables like cell type abundance. With the advancement of single-cell spatial transcriptomic technologies, this is an important work and computational tool that will enable insights. However, the manuscript suffers from a lack of connection to current literature and insufficient methods explanation, limited application of the method, and limited discussion and demonstration of how key parameters and variable affects the overall results to be suitable for publication immediately.

Major concerns:
1. There is very little connection with the host of prior work in the spatial gene expression space. This algorithm should be contextualized better with prior algorithms. Furthermore, the methods section is particularly weak for a paper which describes a new computational approach or method for use in spatial transcriptomics datasets.
2. While there was a demonstration of the algorithm it was quite limited. The authors are in possession of many more datasets. This would allow needed comparisons between tissues, disease and healthy, or across replicates. Understanding the corrected gene expression matrices in these conditions--conditions that will be used by researchers on real-world datasets--will help evaluate how many gene correlation profiles may actually be revealed across the biological heterogeneity across replicates or conditions.
3. There are a number of critical parameters within the presented algorithm. For example, the number of nearest neighbors selected for calculation of gene-coexpression. It is necessary to both provide justification for the values chosen and also show how changing these variables affects overall outcomes of the analysis. Others include but are not limited to subsetting the data for computation and 0.2 correlation threshold.

Minor concern:
The authors state that the correlation of cell types together and associated genes are trivial multiple times; however, these can be biologically informative as well and I would revise such "strong" language around this.

**Authors’ response**

Reviewer #1: The authors propose an innovative solution, InSituCor, to study gene coregulation by regressing out the predominant source of spatial variation, cell types. The flexibility of the approach allows additional covariates to be regressed out as needed. Using the proposed approach, the authors show how phenotypes associated with tumour promotion are detected de novo, highlight the potential of the method in discovering novel molecular mechanisms. The growing plex of spatial technologies will require such tools to enable exploration of molecular phenotypes beyond cell types. Despite the potential of this method, I feel the comments below need to be addressed to ensure users are more informed on how best to apply the method. My main concern is with the lack of details in the methodology reported. I also feel the assumption of normality is unwarranted as the distribution of the data can be modelled using existing statistical tools. The authors need to address this or provide ample evidence supporting the validity of the assumption. After my comments are addressed, the proposed methods will empower spatial biologists to hone in on subtle molecular phenotypes.

> We would like to thank the reviewer for helpful comments and promising suggestions to make InSituCor even more informative. Per the reviewer’s comments, we have substantially expanded our methods section, added a new analysis of tertiary lymphoid structures in the colorectal cancer data, and added several supplementary figures.

Please see our point-by-point response below for details.

Major comments:

1. The methods section is lacking in detail.

> Very true. The editors have suggested we rewrite this as a Methods paper instead of a Short Report. This new format has created room for a much more detailed methods section.

a. Though the methodological detail of InSituCor is provided to a certain extent, details regarding the preprocessing of the data (QC, normalisation, cell typing, …) are missing. Without these, readers are not likely to be able to reproduce the results in this manuscript, nor figure out how they should preprocess their own data to best work with InSituCor.

> We now provide all necessary detail on data pre-processing, although the pipeline is surprisingly simple. The relevant new material states:

“Analysis began with the counts matrix and cell metadata flat files exported by AtoMx. Single cell expression profiles were normalized by dividing each cell’s expression profile by its total counts. Cell types were defined using the Insitutype R package’s semi-supervised mode to fit de novo clusters while also finding known cell types based on reference profiles of immune cells and of mesenchymal cells.”

b. The comprehensive list of variables regressed out and how these are computed also needs to be clarified in the methods. Specifically, I would want to know how the background intensity (library size) is defined as these affect the covariances.

> The methods now note our InSituCor example “conditioned on neighborhood cell type proportions, mean neighborhood total counts per cell, and mean neighborhood negative control probe counts per cell.”

b. (continued) The field of view (FOV) is known to be a batch effect in InSitu spatial technologies. It would be good to know whether this effect is present and whether it has been regressed out as well.

> Good question. FOV artifacts can heavily influence insitucor results, leading to modules that light up impacted FOVs. We consider this a feature rather than a bug, as it makes InSituCor a highly sensitive tool for detecting FOV effects missed during the early stages of analysis. We now note this behavior in the discussion. Our example data was chosen to omit such FOVs.

c. The authors also mention that the module score is computed using a weighted average of genes in the module. The weights used should be specified more explicitly (I assume these are somehow based on the correlation strengths).

> The expanded Methods section now states:

“To mitigate the heteroscedasticity found in genes of varying expression levels, InSituCor’s default uses inverse square root weighting to account for the Poisson-like mean-variance relation seen in count data, defining ¬wj = mean( Y_(∙,j)^((N)))-0.5”. In other words, we use weights to give high- and low-expressing genes appropriate influence, but we do not consider correlation strength.

d. The definition of cell type attribution scores is unclear to me. It would be easier to understand how this is computed if it is defined clearly using mathematical notation.

> Good suggestion. We have added mathematical notation throughout the Methods section.

2. The authors assumed normality and s uggested that averaging out the expression of 50 cells will result in normality due to the central limit theorem. However, if the counts of genes within these individual cells is relatively low (which is the case with most in situ technologies), averaged counts will also be very low and will therefore start resembling a Gamma distribution rather than a normal distribution. In such a setting, instead of simplifying the correlation estimate through a normality assumption, it would be better to fit a generalised linear model with a Poisson (if summing counts), or Gamma family (as means will still have to be positive), and then compute covariances. If the authors still believe that a normal approximation is valid, there needs to provide additional data (possibly in the form of supplementary figures), supporting this claim or showing to what extent normality is true (i.e., is it only valid for highly expressed genes?).

> This is a point worth exploring more deeply. We understand the above to be questioning whether instead of taking conditional covariance with cov(y|x) = cov(y) – cov(y,x)cov(x)-1¬-cov(x,y) we should instead, for each pair of genes, use poisson or gamma glm to model: gene1 ~ gene2 + confounders, then report the partial correlation of gene1 and gene2. Poisson regression won’t work for us, since we take normalized data with non-integer values, but gamma regression will. We implemented this approach and compared it to InSituCor. Unfortunately, fitting pairwise gamma glm’s over thousands of genes would come with a heavy computational cost. Fortunately, we find that our Gaussian-based approach and the alternative gamma glm approach produce very similar results. Please see Supplementary Figure 1 for details.

3. Neighbourhoods for the spatial correlation are defined as the 50 nearest cells to each cell (this should be mentioned in the results section as well). Neighbourhoods based on nearest neighbours will differ in size for dense and sparse regions. The authors should discuss the implications of neighbourhoods defined as such in their discussion section (i.e., will this mean networks are more likely to represent sparse or dense regions?). This should be studied, and guidelines provided to users on what to expect if working with tissues where the cell density varies greatly. Theoretically, spatial correlations are subject to the distances (radius around cells) and could degrade beyond certain distances (this is the basis behind cross-type spatial functions such as the L-cross function). Alternatively, neighbourhoods could be defined by using a fixed radius (or contributions could be weighted softly using a Gaussian function, however, this could complicate the resultant statistics).

> Interesting and complicated point. The Discussion now states:

“Most InSituCor operations are performed on data from cellular neighborhoods, with each cell’s neighborhood defined to capture the set of cells proximal enough to influence it. We prefer the K-nearest neighbors approach to radius-based neighborhoods for the theoretical reason that tissue regions packed densely with cells could be less permeable to cell-cell communications, and for the practical reason that radius-based neighborhoods in sparse tissue regions can contain so few cells that their neighborhood expression becomes statistically unstable. Undoubtedly, the full complexity of tissue biology certainly beggars the K-nearest and radius-based neighborhood concepts implemented within InSituCor: genes and cell types vary in their functional reach, and tissue structures like epithelial walls impede cell and molecule trafficking. Thus the cellular neighborhoods we measure are noisy proxies for true neighborhoods, and this noise likely attenuates InSituCor’s conditional correlation estimates. The problem of optimally defining cellular neighborhoods has not been well- studied. Should better frameworks emerge, InSituCor accepts user-defined cellular neighborhoods.”

The Methods section now notes:

“Neighbors can be defined either with a fixed radius of as the K-nearest cells. Recognizing that both these concepts of “neighborhoods” are limited, InSituCor also accepts custom-defined cell neighborhoods, making it forward-compatible as more advanced neighborhood definitions enter the literature.”

Finally, the new Supplementary Figure 3 addresses the impact of neighborhood definition on the conditional correlation matrix.

4. Gene regulation is context specific with the context potentially being a spatial domain. We would expect different regulatory networks to be active in tumour regions compared to surrounding normal tissue. The proposed approach will mask correlations that are present only in a small region of the tissue. An extreme example of this would be when genes are co-expressed in a small region, but randomly expressed else where (or either one of the genes is primarily expressed elsewhere). As correlation is measured globally, these local associations would be missed. It will enhance the method if the authors allow users to define domains and run the analysis within domains to obtain domain-specific networks. This can be addressed by simply explaining in text this possibility and emphasising that users should then perform analysis within each domain. Alternatively, the method can be designed to take domain as an input and produce domain-specific networks (while potentially highlighting edges that are common across domains).

> Good idea. We explored the concept of niche-specific networks by re-running InSituCor only within the tertiary lymphoid structures in the colorectal tumor. This turned out to have two benefits: first, it produced results focused on the selected biology, helping interpretation. Second, confirming your hypothesis above, it identified 52 strong gene-gene correlations missed by the dataset-wide analysis. Figure 2e,f and Supplementary Figure 7 show results of this analysis. The results now state, “The TLS- only analysis returned 52 gene pairs with strong (>0.5) conditional correlations that had been near-zero (<0.1) in the whole-tissue analysis, suggesting that a spatially-targeted approach can discover relationships obscured in tissue-wide analyses.”

The idea of contrasting networks from different domains is also interesting. In the new Figure 3 we undertake a closely-related exercise, contrasting networks from different tissues. The Discussion now notes, “Our application of InSituCor to only the tertiary lymphoid structures in the colon tumor showed that networks focused on biology of interest can be more informative. A straightforward extension would be to compare networks found in contrasting spatial domains, for example tumor interior vs. tumor border. The approach for multi-sample analysis in Figure 3 could apply directly to the domain-specific problem: InSituCor could be run separately across multiple spatial domains, and conditional correlations can be compared directly.”

5. The authors sub-sample the data (5000 cells) to estimate the correlations. Given that the sampling rate is very low (~4%), the authors need to provide supplementary results to demonstrate the stability of estimates across repeated random samplings at this rate and at higher rates. It is important for users to know how the effect of sampling rates vary and what they should expect as they trade off accuracy for computing times. I suspect the estimates would be reliable but it will be good to see evidence of this.

> Good point. The new Supplementary Figure 2 explores the impact of subsample size on the conditional correlation matrix.

Minor comments:

1. Panels c-f and l-m in figure 1 need axis titles to make it clear what is being plot.

> We have added axis labels where they were missing from these figures.

2. Legends for the heatmap and spatial plots (1l, 2c-e) are missing in both figures. It is important to know the scales of these values and whether they have been rescaled for visualisation purposes.

> The figure legend for 1i and the legend for 2c-e now both state, “color scale ranges from 0 expression to the 95th percentile of the module’s expression.”

3. It would help to have heading for some panels to make it easier to read the figures on their own. For instance, 1i could have the heading (or legend title) "Tumour- promoting inflammation score", that makes it easy to infer what is plot. Likewise, for the heatmaps.

> Good idea. We have done this.

4. The first sentence in the abstract is too open-ended and can be trimmed out to emphasise on the problem that InSituCor is designed to address.

> We have removed this sentence and revised the abstract.

5. By formally defining two matrices, the environment matrix (say Y), and the covariate/confounder matrix (X), equation 1 and 2 can be combined to provide a more concise definition.

> We now include this formal definition in the Methods. For the sake of readability, we have elected to keep equation (1) as-is to minimize the reader’s cognitive burden while becoming familiar with the concepts.

6. The correlation analysis performed by InSituCor can possibly shed light on regulatory associations. I am curious to see how many of the nodes are known human transcription factors and where these are the central nodes in the network obtained by InSituCor. Besides a module-based analysis, central nodes (with higher degree centrality) could be an independent means to identify regions in the tissue where a known transcription factor is active. If the authors identify any such genes, it would be a strong demonstration to an alternative approach to interpret biology using InSituCor.

> We explored this concept by using igraph’s implementation of Kleinberg’s hub score within each module. Somewhat surprisingly, for almost all modules, the top two-ranking hub genes matched identically the two genes InSituCor selected to name the module. (InSituCor names modules by the genes with the highest within-module conditional

correlation.)

We also tried selecting nodes based on the entire conditional correlation matrix. However, in this analysis, all the top-scoring genes came from the largest module, so we discarded this approach.

The transcription factor idea is appealing. Unfortunately, the CosMx 6000-plex panel doesn’t include many transcription factors, as they tend to have low expression.

7. Rather than manually inferring tumour promoting inflammation from the 17 genes in the modules, the authors could use gene-set enrichment analysis (with the 6000 genes in the panel as background) to identify common processes represented by their module using existing tools such as vissE (PMID: 38331751) or EnrichmentMap (PMID: 30664679).

> This is a promising idea. We used gene set enrichment analysis of Reactome pathways to describe our module of interest. The three most-enriched pathways were “Initial triggering of complement”, “Common Pathway of Fibrin Clot Formation”, and “Activation of Matrix Metalloproteinases”; however, none of these pathways was impressively enriched in the module. We now discuss the possibility of this approach and others in the Discussion.

8. R-based bioinformatics tools are often more widely adopted when submitted to Bioconductor (or CRAN). I would advise the authors to attempt this submission to improve dissemination. It also ensures that the package works seamlessly with other Bioconductor packages. Detailed instructions can be found at https://contributions.bioconductor.org/bioconductor-package-submissions.html. Bioconductor staff are very helpful and will guide on how compliance can be achieved during peer-review.

> Unfortunately, CRAN and Bioconductor do not accept the non-standard open-source software license that our legal department insists on. To ensure compatibility with diverse packages and workflows, InSituCor accepts base R objects (matrices, vectors) rather than the highly structured objects favored by e.g. the Seurat and Bioconductor communities. InSituCor’s necessary inputs are straightforward to extract from these objects.

Reviewer #2: Danaher et al. created a pipeline to look at spatial gene correlations while removing variables like cell type abundance. With the advancement of single-cell spatial transcriptomic technologies, this is an important work and computational tool that will enable insights. However, the manuscript suffers from a lack of connection to current literature and insufficient methods explanation, limited application of the method, and limited discussion and demonstration of how key parameters and variable affects the overall results to be suitable for publication immediately.

> We would like to thank the reviewer for their time, effort and helpful comments. To address these comments, we have discussed the existing literature in greater depth, substantially expanded our methods section, added an analysis of an additional dataset of pancreatic tumors, and added several supplementary figures. Please see our point-by-point response below for details.

Major concerns:

1. There is very little connection with the host of prior work in the spatial gene expression space. This algorithm should be contextualized better with prior algorithms. Furthermore, the methods section is particularly weak for a paper which describes a new computational approach or method for use in spatial transcriptomics datasets.

> These are both fair points. The editors have suggested we rewrite this as a Methods paper instead of a short report. This new format has allowed much more extensive discussion of prior work and of detailed methods.

The background now discusses prior work as follows:

“Recognizing the promise of spatially-varying expression patterns to highlight noteworthy biology, researchers have proposed diverse methods for identifying single genes with spatially auto-correlated expression patterns, and for the related problem of identifying sets of genes that are spatially correlated with each other. SpatialDE (CITE), the first method designed for this purpose, models spatial expression with a Gaussian process. Trendseek (CITE) uses point processes, a mainstay from the field of spatial statistics. scGCO (CITE) fits hidden Markov random fields. MaxSPIN (CITE) employs machine learning to approximate mutual information between nearby expression measurements. SpatialDM (CITE), which focuses on ligand-receptor pairs, uses a modified Moran’s I statistic. SpaGFT (CITE) performs a spatial Fourier transformation to analyze gene expression in the frequency domain rather than the spatial domain. Similar to the method we propose, SPARK-X (CITE) takes a non-parametric, kernel- based approach. Pursuing a slightly different goal, DIALOUGE (CITE) uses penalized estimation to infer “multi-cellular programs”. In addition, legacy methods from the field of spatial statistics, in particular Moran’s I (CITE) and Lee’s L (CITE), have gained wide use.”

The Methods section has been expanded throughout.

2. While there was a demonstration of the algorithm it was quite limited. The authors are in possession of many more datasets. This would allow needed comparisons between tissues, disease and healthy, or across replicates. Understanding the corrected gene expression matrices in these conditions--conditions that will be used by researchers on real-world datasets--will help evaluate how many gene correlation profiles may actually be revealed across the biological heterogeneity across replicates or conditions.

> Good suggestion. To demonstrate multi-sample workflows, we have added an additional analysis of 19 pancreatic cancers, shown in the new Figure 3 and in Supplemental figures 8-11. We first explore correlations found consistently across samples, building a consensus correlation network. Then, we identify correlations found in only subsets of samples, discovering gene modules activated in different cancer morphologies. Finally, we contrast correlations found in two classes of samples. This analysis of network differences across samples demonstrates an important additional use case for InSituCor.

3. There are a number of critical parameters within the presented algorithm. For example, the number of nearest neighbors selected for calculation of gene- coexpression. It is necessary to both provide justification for the values chosen and also show how changing these variables affects overall outcomes of the analysis. Others include but are not limited to subsetting the data for computation and 0.2 correlation threshold.

> Good point. We now address the impact of our tuning parameters in Supplementary Figures 2-5.

In addition, for a detailed discussion of the definition of cellular neighborhoods, see the new Discussion section and our response to Reviewer 1, point 3.

Minor concern:

The authors state that the correlation of cell types together and associated genes are trivial multiple times; however, these can be biologically informative as well and I would revise such "strong" language around this.

> We have reworded accordingly.

**2^nd^ round**

**Reviewer 1**

The authors have addressed all my concerns by revising the text and performing numerous additional experiments. They have included all my recommendations as part of the revised manuscript. The only minor concern I have is regarding the layout of the figures. The panelling seems crude and not aesthetic as per the standards of articles published in Genome Biology. I would recommend the authors to align the panels well, aligning text and legends where possible, and aligning the labels for panels. I am happy for the editorial team to assess this after any updates and progress without my inclusion.

**Reviewer 2**

I appreciate the authors addressing all of my concerns.
